# Correlation between HIV viral load and aminotransferases as liver damage markers in HIV infected naive patients: a concordance cross-sectional study

**DOI:** 10.1186/1743-422X-6-181

**Published:** 2009-10-30

**Authors:** José Antonio Mata-Marín, Jesús Gaytán-Martínez, Bernardo Horacio Grados-Chavarría, José Luis Fuentes-Allen, Carla Ileana Arroyo-Anduiza, Alfredo Alfaro-Mejía

**Affiliations:** 1 Infectious diseases department, Hospital de Infectología, "La Raza" National Medical Center, IMSS, Mexico City, México; 2 Internal medicine department, Hospital de Especialidades, "La Raza" National Medical Center, IMSS, Mexico City, México; 3 Clinical pathology department, Hospital General, "La Raza" National Medical Center, IMSS, Mexico City, México

## Abstract

Abnormalities in liver function tests could be produced exclusively by direct inflammation in hepatocytes, caused by the human immunodeficiency virus (HIV). Mechanisms by which HIV causes hepatic damage are still unknown. Our aim was to determine the correlation between HIV viral load, and serum levels of aspartate aminotransferase (AST) and alanine aminotransferase (ALT) as markers of hepatic damage in HIV naive infected patients.

We performed a concordance cross-sectional study. Patients with antiviral treatment experience, hepatotoxic drugs use or co-infection were excluded. We used a Pearson's correlation coefficient to calculate the correlation between aminotransferases serum levels with HIV viral load. We enrolled 59 patients, 50 men and 9 women seen from 2006 to 2008. The mean (± SD) age of our subjects was 34.24 ± 9.5, AST 37.73 ± 29.94 IU/mL, ALT 43.34 ± 42.41 IU/mL, HIV viral load 199,243 ± 292,905 copies/mL, and CD4+ cells count 361 ± 289 cells/mm3. There was a moderately strong, positive correlation between AST serum levels and HIV viral load (r = 0.439, P < 0.001); and a weak correlation between ALT serum levels and HIV viral load (r = 0.276, P = 0.034); after adjusting the confounders in lineal regression model the correlation remained significant. Our results suggest that there is an association between HIV viral load and aminotransferases as markers of hepatic damage; we should improved recognition, diagnosis and potential therapy of hepatic damage in HIV infected patients.

## Introduction

HIV-AIDS is one of the main causes of mortality over the world; during the last decade the amount of human immunodeficiency virus (HIV) infected patients has increased dramatically worldwide [1-3].

In HIV infected patients, the increase in hepatic enzymes could be secondary to multiple factors such as alcoholism, lipid lowering drugs, co-infection with hepatitis viruses, or hereditary diseases; in addition, it has been proposed that HIV causes a direct damage over hepatic cells [4-9]. Many factors are associated with hepatic damage: antiretroviral treatment, co-infections with hepatitis B or C virus, opportunistic infections as citomegalovirus, mycobacterium, leishmaniasis, or tumors (lymphoma and Kaposi's sarcoma), cholangitis associated to parasites (cryptosporidiosis and microsporidiosis) and toxicity related with non antiretroviral drugs (trimetoprim and other antibiotics)[10].

Abnormalities in liver function tests could be produced exclusively by direct inflammation in hepatocytes, caused by the virus. Mechanisms by which HIV causes hepatic damage are still unknown, but the most important mechanisms could be apoptosis (induced by caspases 2, 7 and 8) and mitochondrial dysfunction with decreasing in mitochondrial DNA in several tissues; another injury mechanism is permeability alteration in mitochondrial membrane by HIV proteins which stimulate an inflammatory response [9-16].

Aspartate aminotransferase (AST) and alanine aminotransferase (ALT) are hepatic enzymes that could be used as markers of hepatocellular injury [17].

The purpose of the study was to determine the correlation between HIV viral load, with serum levels of AST and ALT as markers of hepatic damage in HIV naïve infected patients.

## Patients and methods

We performed a concordance cross-sectional study in which HIV infected naïve patients without opportunistic or co-infections at the moment of the evaluation were included. We enrolled patients seen at the Hospital de Infectología, "La Raza" National Medical Center in Mexico City from January 2006 to September 2008. Patients with recent diagnosis of HIV were eligible for the study if they were between the ages of 18 and 65 years old with negative serology to hepatitis B or C. Patients were excluded if they had alcohol consumption during the last three months or had suffered any kind of opportunistic disease or co-infection.

We ascertained patients' general attributes (age, sex), medical history, risk factors, medications, height, weight, vital signs, HIV infection confirmed by ELISA and Western-blot; and the following laboratory tests: blood cells count, bleeding times, blood chemistry, liver function tests and CD4+ cells count were obtained. In addition TORCH panel and VDRL were requested; HIV viral load was measured using reverse-transcriptase polymerase chain reaction (RT-PCR) test, with a detection limit lower than < 50 copies/ml.

Medical history and physical examination were performed in order to exclude any kind of opportunistic infection or definitory AIDS disease.

The strength of relationship between HIV viral load and AST and ALT was estimated by a Pearson correlation coefficient. To adjust for the effects of potential confounders, we used a linear regression model. The statistical significant difference was considered when p-value was less than 0.05.

## Results

We enrolled 59 recent diagnosis HIV naïve infected patients; 84.7% (n = 50) of whom were men; and the mean (± SD) age was 34.24 ± 9.54 years; regarding sexual preferences, about men 68% (n = 34) were men who have sex with men; mean men sexual partners was 17.9 ± 45.4 couples and women 1.8 ± 1.1 couples. Half of male patients, 25 of 50, had ALT levels 30 IU or more per milliliter at baseline; and most female patients, 6 of 9 had ALT levels 19 IU/ml or more. Mean HIV viral load was 199,243 ± 292,905 copies/ml and CD4+ cells count 361 ± 289 cells/ml. Mean (± SD) cholesterol was 143.64 ± 31.45 mg/dl and triglycerides was 164.22 ± 94.90 mg/dl. Baseline characteristics were recorded (Table 1).

**Table 1:** Basal characteristics of 59 HIV naïve infected patients

**Variable**	**Minimum**	**Maximum**	**Mean**	**SD**
Age (years)	20	61	34.24	9.54
Mass corporal index (kg/m^2^)	17.7	31.45	24.32	4.90
Sexual partners number	1	300	17.92	45.44
Hemoglobin g/dl	8.2	18.2	14.81	1.86
Platelets × 10^-9^/liter	60,100	350,000	215,747	51,851
Leucocytes × 10^-9^/liter	2.2	11	5.59	1.79
Glucose mg/dl	73	115	91.47	9.52
Creatinine mg/dl	0.7	1.4	0.95	0.14
Cholesterol mg/dl	68	220	143.64	31.45
Triglycerides mg/dl	57	450	164.22	91.90
Albumin g/dl	3.2	5.5	4.63	0.54
AST IU/mL	13	158	37.73	29.94
ALT IU/mL	12	276	43.34	43.34
CD4+ cells/mL	9	1495	361.42	289.56
HIV viral load (copies/mL)	500	1,000,000	199,243	292,905

### Correlation of HIV viral load with AST and ALT

**Figure 1:**
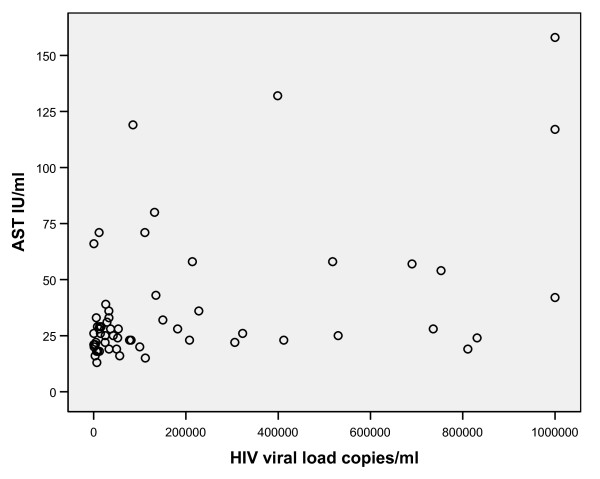
**Scatter plot of HIV viral load and AST**. There is a significant moderately strong, positive correlation between HIV viral load and AST (r = 0.439, P = 0.001).

**Figure 2:**
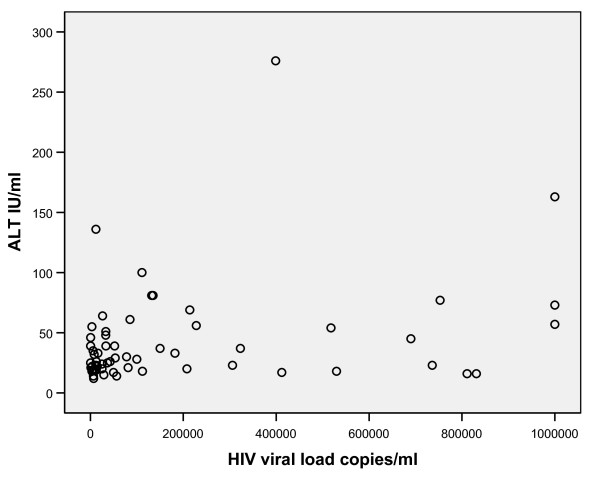
**Scatter plot of HIV viral load and ALT**. There is a significant mild strong, positive correlation between HIV viral load and ALT (r = 0.276, P = 0.034).

There was a significant moderately strong, positive correlation between HIV viral load and AST (Pearson correlation coefficient = 0.439, P = 0.001) (Figure 1) there was also a significant mild strong, positive correlation between HIV viral load and ALT (Pearson correlation coefficient = 0.276, P = 0.034) (Figure 2).

Adjusting for age, cholesterol, triglycerides and CD4+ cells count in a multivariable linear regression model did not affect the results. HIV viral load and AST remain significant (p = 0.002) either do ALT (p = 0.021)

## Discussion

Our results suggest the possibility that in HIV naïve infected patients, liver injury could be the effect of HIV *per se *at least partially. It was found a positive correlation between HIV viral load and aminotrasferases as liver damage markers in naïve patients without any kind of opportunistic diseases or co-infections. There was a stronger positive correlation between AST and HIV viral load than with ALT, probably due to other tissues injury (muscle, lung, and kidney). We explain our results by effect of apoptosis induced by the viral proteins such as Tat, Nef, Vpr, protease and gp120 in different cell groups. These findings support partially the theories of liver damage due to mitochondrial disturbance and the stimulation of the caspases cascade in the induction of apoptosis.

These results indicate that clinicians should consider monitoring aminotransferases levels in all HIV patients including those without antiretroviral experience. Based in our results, it is possible that patients with an increase in aminotransferases could be potentially benefited with HAART.

There are few studies assessing liver injury associated to HIV; most of them are basic designs trying to find molecular mechanisms of liver damage [13-15]; other studies evaluate liver damage induced by the association of hepatitis C virus and HIV.

Our findings are consistent with those of Ejilemele *et al*., reporting common abnormalities of liver enzymes in HIV patients; however, they only describe patterns of liver injury and they didn't establish strength of relationship between HIV viral load and liver enzymes [18].

Most studies that evaluate liver steatosis include patients infected with HCV genotype 3; this genotype has been associated to liver steatosis [19,20].

Sulkowski et al., examined liver tissue from 112 antiretroviral experienced HIV-HCV co-infected patients; 60% had no evidence of histologic liver steatosis, and only 18% had steatosis with more than 5% hepatocytes affected, but they didn't evaluate HIV mono-infected naïve patients [20].

Unfortunately the sample we studied was small; and that increases the possibility of a higher random error. Another limitation is the cross-section design which is unable to measure all potential confounders; however, in analysis, confounding factors were controlled; then, we consider results are valid for our population.

According to our results there is a correlation between the HIV viral load, and aminotransferases serum levels in HIV infected naïve patients. Liver function tests should be monitorized closely in these patients; and we suggest evaluating early start of antiretroviral treatment in HIV infected patients with severe liver damage regardless of CD4+ cell count.

It is necessary to perform a study including more patients, using direct or indirect liver damage measures (biopsy, fibrotest or transient elastography) to improve recognition, diagnosis and management. Finally, future prospective research to study antiretroviral therapy effects on reducing aminotransferases levels in these patients should be developed.

## Competing interests

The authors declare that they have no competing interests.

## Authors' contributions

JAMM, JGM and BHGC performed the majority of experiments. All the authors provided the collection of all the human material in addition to providing financial support for this work; JAMM gave the analytic tools to this paper. JAMM, JLFA, JGM, AAM and CIAA provided vital reagents and were also involved in editing the manuscript. JAMM designed the study and all the authors wrote and approved the final manuscript.
